# In Vitro Evaluation of Surface and Mechanical Behavior of 3D-Printed PMMA After Accelerated and Chemical Aging Under Simulated Oral Conditions

**DOI:** 10.3390/dj14010040

**Published:** 2026-01-07

**Authors:** Vlad-Gabriel Vasilescu, Robert Cătălin Ciocoiu, Andreea Mihaela Custură, Lucian Toma Ciocan, Marian Miculescu, Vasile Iulian Antoniac, Ana-Maria Cristina Țâncu, Marina Imre, Silviu Mirel Pițuru

**Affiliations:** 1 Discipline of Dental Prosthesis Technology, Faculty of Dentistry, “Carol Davila” University of Medicine and Pharmacy, Dionisie Lupu Street, No. 37, District 2, 020021 Bucharest, Romania; vlad.vasilescu@umfcd.ro; 2 Faculty of Materials Science and Engineering, National University of Science and Technology POLITEHNICA Bucharest, Splaiul Independentei 313, J Building, 060042 Bucharest, Romania; ciocoiurobert@gmail.com (R.C.C.); iulian.antoniac@upb.ro (V.I.A.); 3 Discipline of Prosthodontics, Faculty of Dentistry, “Carol Davila” University of Medicine and Pharmacy, 37 Dionisie Lupu Street, District 2, 020021 Bucharest, Romaniamarina.imre@umfcd.ro (M.I.); 4 Department of Organization, Professional Legislation and Management of the Dental Office, Faculty of Dental Medicine, “Carol Davila” University of Medicine and Pharmacy, 17-23 Plevnei Str., 020021 Bucharest, Romania

**Keywords:** 3D-printed PMMA, provisional dental materials, accelerated aging, chemical aging, thermal aging, surface free energy, mechanical properties

## Abstract

Studying surface energy and permeability offers insights into the relationship between temporary polymers and the oral environment. Variations in contact angle and surface free energy may signify modifications in surface polarity and tendency for plaque buildup, staining, or microcrack formation. **Objectives**: The present study aims to evaluate the influence of simulated salivary and chemical aging conditions on the surface and mechanical properties of 3D-printed PMMA provisional materials. **Methods**: Two 3D-printed polymethyl methacrylate (PMMA) resins were investigated, namely Anycubic White (Anycubic, Shenzhen, China) and NextDent Creo (NextDent, 3D Systems, Soesterberg, The Netherlands), using two aging protocols. Protocol A consisted of chemical aging in an alcohol-based mouthwash, while Protocol B involved thermal aging in artificial saliva. After aging, surface properties (wettability and SFE) and compressive behaviour were analyzed. Statistical analysis was conducted to assess the influence of temperature, immersion duration, and aging medium, with significance established at *p* < 0.05. **Results**: In Protocol A, mechanical properties showed a time-dependent decrease, with material-specific stabilization trends. In Protocol B, thermal aging resulted in elastic modulus reductions ranging from 35% to 46% relative to the reference. The yield strength exhibited similar tendencies. In Protocol A, X samples exhibited a consistent decline, while C samples stabilized after 14 days. For Protocol B, the fitted model produced residuals under 2%, confirming temperature as the primary variable. **Conclusions**: Chemical and thermal aging influence the physical and mechanical properties of the analyzed 3D-printed PMMA. Among the two protocols, thermal aging in artificial saliva resulted in more pronounced material degradation. After chemical aging in mouthwash, the surface free energy remained almost constant. After thermal aging, all samples demonstrated a gradual rise in SFE with prolonged immersion duration. The current study offers valuable insights into the environmental stability of printed PMMA; however, it is an in vitro evaluation. The findings indicate that temperature exposure and prolonged contact with oral hygiene products may affect the mechanical reliability of 3D-printed provisional restorations, which must be considered during material selection for longer temporary usage. Additionally, spectroscopic and microscopic analyses might better clarify the molecular-level chemical alterations linked to aging.

## 1. Introduction

Polymethyl methacrylate (PMMA) has been extensively utilized for temporary crowns and bridges due to its appealing aesthetics, ease of manipulation, and favorable biocompatibility [1]. In modern restorative dentistry, PMMA is offered in many processing forms—conventional autopolymerized, heat-polymerized, CAD/CAM milled, and 3D-printed resins—each demonstrating unique mechanical and surface properties [2,3]. The advent of subtractive and additive manufacturing has significantly improved the precision and consistency of temporary restorations; yet, uncertainties persist concerning their long-term performance under the variable chemical and temperature conditions of the oral cavity [4].

Provisional restorations in the intraoral environment are consistently exposed to humidity, temperature changes, and alterations in salivary pH [5]. Salivary pH may diminish owing to dietary practices, gastric reflux, or inadequate oral hygiene, while the regular use of alcohol-based mouth rinses introduces an additional chemical stressor that may alter polymer structure [6,7]. These environmental conditions may facilitate water absorption [5,8], deformation, or surface softening [9], resulting in alterations to the material’s roughness [10], color stability [11], and mechanical properties [12]. Despite their clinical significance throughout the relatively short lifespan of temporary restorations, these effects are frequently overlooked in material selection and laboratory evaluation [13].

Recent investigations indicate that PMMA-based materials exhibit different resistance to pH-induced degradation, depending on the polymerization technique and degree of conversion. Lahoti et al. [6,7] noted that acidified saliva significantly decreased the flexural and impact strength of heat polymerized PMMA specimens, indicating that chemical aging can undermine structural integrity even after brief exposure durations. Conversely, CAD/CAM-milled PMMA blocks, produced at high pressure and temperature, often demonstrate reduced residual monomer content and better dimensional stability relative to traditionally cured materials [14,15]. Still, the newly developed class of 3D-printed PMMA resins, while favorable to digital processes, has been shown to exhibit increased sorption, greater surface roughness, and diminished hardness upon immersion in water or alcohol-based solutions [16].

Studying surface energy and permeability offers vital insights into the relationship between temporary polymers and the oral environment. Variations in contact angle and surface free energy may signify modifications in surface polarity and predict the tendency for plaque buildup, staining, or microcrack formation. Accelerated aging experiments utilizing artificial saliva or mouthwash immersion provide significant insight into the chemical durability of 3D-printed and milled PMMA crowns.

Despite expanding research on the aging behavior of 3D-printed PMMA materials, most existing studies have concentrated on singular aging settings and a restricted range of material attributes. Numerous studies have assessed chemical aging by immersion in artificial saliva, water, or alcohol-based mouthwashes, focusing mainly on water absorption, surface roughness, color stability, or specific mechanical properties [17,18]. In contrast, alternative research has investigated thermal or thermo-cycling procedures, primarily evaluating flexural strength, hardness, or dimensional stability under temperature-induced stress [19,20].

Even so, these methodologies are often executed in isolation, employing distinct experimental designs, aging periods, and assessment endpoints, thereby limiting direct comparisons of deterioration mechanisms. Furthermore, surface-related metrics and bulk mechanical behaviour are frequently examined independently, despite their collective significance for the clinical efficacy of interim restorations.

Currently, direct comparison data that concurrently evaluate the effects of chemical and thermal aging on both surface properties and mechanical performance of 3D-printed PMMA within an integrated experimental framework are limited. Understanding this shortcoming is crucial for considering how various intraoral stresses influence material deterioration and for facilitating the selection and use of 3D-printed PMMA provisional materials.

This study employs a comparative dual-protocol approach, contrasting immersion-based aging with thermal aging within a unified experimental framework to evaluate the efficiency of aging protocols and their relationship with the degradation of mechanical properties, in contrast to prior investigations that focused on isolated aging protocols.

The hypothesis tested in this study was that chemical and thermal aging protocols would differentially impact the surface and mechanical properties of 3D-printed PMMA materials, with thermal aging resulting in more significant mechanical degradation and chemical aging predominantly affecting surface characteristics.

The present study aims to evaluate the influence of simulated salivary and chemical aging conditions on the surface and mechanical behavior of 3D-printed PMMA provisional materials. By analyzing contact angle variations, surface free energy, hardness, and compressive characteristics after exposure to artificial saliva at different temperatures and to alcohol-containing mouthwash, this research seeks to elucidate changes in material resistance. The findings are expected to guide the clinical selection of provisional materials and support the optimization of additive manufacturing parameters for increased intraoral performance.

## 2. Materials and Methods

### 2.1. Materials

Two 3D-printed polymethyl methacrylate (PMMA) resins were investigated: Anycubic Basic White (Anycubic, Shenzhen, China), coded X, and NextDent Creo (NextDent, 3D Systems, Soesterberg, The Netherlands), coded C. Two commercially accessible 3D-printed PMMA resins were chosen to exemplify distinct kinds of materials often utilized for temporary restorations. Anycubic PMMA is a commonly available resin utilized in desktop additive manufacturing, whereas NextDent Creo PMMA is a dental-certified material particularly designed for clinical usage.

Despite both materials being PMMA-based, variations in formulation, polymerization degree, and intended therapeutic application are anticipated to affect their aging response. The comparative analysis of these two materials allows the determination of whether accelerated chemical and thermal aging effects depend on the material and offers insight into the performance heterogeneity among frequently utilized 3D-printed PMMA resins.

Both materials were printed in accordance with the manufacturer’s specifications and subsequently post-cured using light polymerization units under standardized conditions. Rectangular (10/10/10 mm) flat specimens were fabricated to facilitate surface and mechanical testing.

### 2.2. Experimental Design

The degradation of 3D-printed provisional PMMA restorations under intraoral-like conditions was simulated using two complementary in vitro experimental protocols.

Unaged specimens were provided for each material for reference purposes. The as-printed Anycubic PMMA specimens were designated as XR, whereas the as-printed NextDent Creo specimens were designated as CR. These reference groups served as baseline controls for comparison with aged specimens.

Protocol A (chemical aging) evaluated the effect of prolonged exposure to an alcohol-based mouthwash, with the goal of assessing the impact of extended immersion in an ethanol-containing solution on surface and mechanical characteristics. Chemical aging was conducted with a singular commercially available alcohol-based mouthwash as a standardized chemical challenge. This method was deliberately chosen to provide experimental control and repeatability, facilitating the independent evaluation of solvent-related impacts on 3D-printed PMMA materials.

The oral environment involves several chemical exposures; nonetheless, employing a singular mouthwash solution serves as a typical and often utilized in vitro model for assessing the impact of chemical aging, as documented in prior dental materials research. The current methodology was not designed to reproduce the complete chemical intricacy of the mouth cavity, but instead to facilitate a controlled comparison between materials under uniform exposure settings. The specimens were entirely submerged in a commercial mouthwash at 37 °C, simulating intraoral temperature. Exposure durations were established at 7, 14, 21, and 28 days, with the immersion medium renewed every seven days to preserve uniform chemical composition. Each specimen was assigned a code based on the printing substance (X = Anycubic; C = NextDent) and immersion period (7–28 days, e.g., X7, C14, or C28).

Protocol B (thermal aging) assessed the influence of temperature and exposure period in artificial saliva on surface wettability and surface free energy. The printed specimens were submerged in artificial saliva and kept at regulated temperatures of 40 °C, 50 °C, and 60 °C for 48, 60, and 72 h, respectively. Each experimental group was designated by a code that combines temperature and exposure duration (for instance, 4048 signifies 40 °C for 48 h). These parameters (time and temperature) were used in a 2^2^ factorial design to see the contribution of each factor in an accelerated aging experiment. The specified thermal aging values were established based on previously documented accelerated aging techniques for dental polymeric materials [21]. Temperatures ranging from 40 °C to 60 °C [22] have been extensively employed to replicate heightened intraoral thermal stress while keeping within the glass transition temperature of PMMA (which is between 85 and 165 °C), thereby preventing irreversible thermal deterioration. The upper temperature limit was set at 60 °C to ensure sufficient thermal acceleration while avoiding proximity to the glass transition temperature (Tg) of PMMA [23]. The lower temperature limit of 40 °C was selected as an approximation of physiological intraoral temperature, rounded for experimental simplicity. Maintaining temperatures well below Tg minimizes the risk of irreversible thermal damage unrelated to clinically relevant aging mechanisms. A central experimental point (50 °C) was included to verify the assumed planar behavior of the response surface and to assess the adequacy of the regression model. This approach enabled efficient analysis of factor influence while limiting the total number of experimental runs. Exposure periods of 48 to 72 h were chosen to induce measurable aging effects within a feasible study period. Accelerated aging models suggest that brief exposure to high temperatures might correspond to extended clinical treatment durations under physiological settings [24]. Comparable heat ranges and durations have been utilized to assess the long-term performance of polymer-based dental materials, such as PMMA, in simulated oral conditions.

These two complementary protocols offered a thorough assessment of PMMA behavior under simulated clinical aging conditions—thermal stress corresponding to accelerated environmental exposure and chemical stress suggesting a prolonged interaction with alcohol-based oral hygiene products.

### 2.3. Artificial Saliva Preparation

Artificial saliva was prepared using a Fusayama-type formulation by dissolving sodium chloride, potassium chloride, calcium chloride, sodium dihydrogen phosphate, sodium sulfide, and urea in deionized water under continuous magnetic stirring. The complete composition and concentrations are provided in Table 1 [25]. After complete dissolution, the final volume was adjusted to 1 L, and the pH was regulated to 5.2 using diluted sodium hydroxide or hydrochloric acid solutions. The solution was freshly prepared prior to use to ensure chemical stability [5].

All solutions were substituted weekly to ensure chemical stability and prevent contamination.

### 2.4. Surface Characterization

Both protocols were conducted using the same surface characterization process.

Surface wettability was assessed with a KRUESS DSA30 goniometer (Kruess GmbH, Hamburg, Germany) by the sessile-drop technique. Contact angles were determined using three liquids—deionized water, diiodomethane, and ethylene glycol—five measurements per specimen were performed, and the average value was calculated.

The surface free energy (SFE) was determined using the Fowkes, Owens–Wendt–Rable–Kaelble (OWKR), Wu, and Girifalco–Good methods [26,27,28,29,30,31,32]. This is because each method uses a different assumption. The Fowkes method assumes that the adhesion and surface free energy are induced from dispersion, van der Waals forces. The OWKR method divides the surface free energy into dispersive and polar components, adding complexity to the possible interactions. The Wu method is a modified version of OWKR and can provide improved results for complex surfaces or different interaction behavior. The Girifalco–Good method is mainly applied to study the interfacial energy of solids and liquids, and it uses a material parameter in its estimation. A combined use of these methods should provide insight into severe surface changes, given their theoretical assumptions.

### 2.5. Mechanical Characterization

In Protocol A, the mass variation (Δw %) was also determined by weighing the specimens before and after immersion using a precision balance (±0.001 g).

Mechanical testing was performed just on the specimens undergoing the chemical aging process, while the thermal experiment sought to assess surface-related phenomena exclusively. The Shore D hardness of each specimen was assessed using a manual durometer, doing five independent measurements per sample and determining the average to provide a representative result for each experimental condition.

To determine the compressive behavior, evaluations were conducted utilizing a Walter + Bai LFV 300 universal testing machine, which was run under displacement control at a constant crosshead velocity of 5 mm/min. Throughout the testing, force–displacement curves were consistently documented and then analyzed to investigate the material’s distinctive properties, including the elastic modulus, yield strength (defined at 0.2% offset strain), and the thresholds of elastic and plastic deformation. The tests provide quantitative insights into the structural integrity and mechanical stability of the aged PMMA materials under simulated intraoral circumstances.

### 2.6. Statistical Analysis

All quantitative data were analyzed with SPSS v25 (IBM Corp., Armonk, NY, USA).

Analysis of variance (ANOVA) was utilized to determine statistically significant impacts of temperature, immersion duration, and medium. Post hoc Tukey and Fisher tests were utilized to evaluate the equality of group means.

Statistical significance was established at *p* < 0.05 for all analyses.

A literature-based analysis was performed to determine the deterioration of the mechanical properties of polymethyl methacrylate (PMMA) during immersion aging in fake or natural saliva. Most existing investigations focused on the temporal fluctuation of compressive strength, with immersion durations generally categorized at 1, 3, 6, and 12 months.

To reduce the impact of intrinsic material characteristics, the progression of mechanical behavior was evaluated by the % decrease in compressive strength compared to unaged reference specimens, as per the following relation [5,33,34,35,36]:

$$\%D=\frac{\sigma_{r}-\sigma_{t}}{\sigma_{r}}\times 100$$

where

- Δσ represents the percentage decrease in compressive strength;
- σ_0_ is the compressive strength of the unaged reference specimen (MPa);
- σ_t_ is the compressive strength of the specimen aged for time t (days).

The sample size (n = 3 per experimental condition) was determined as the smallest number of duplicates necessary to get meaningful statistical data while ensuring experimental feasibility. The significant repeatability that comes with additive manufacturing methods, evidenced by the minimal variability in the experimental outcomes, justifies the utilization of duplicate specimens for destructive mechanical testing.

For compressive testing, which is inherently damaging, three specimens per condition were considered adequate to assess experimental variability and provide comparative statistical analysis within a structured experimental framework. Conversely, contact angle and Shore D hardness measurements were non-destructive and conducted repeatedly on the lateral surfaces of each specimen, facilitating a greater number of determinations per sample and enhancing measurement robustness.

Response surface methodology (RSM) was chosen for its capacity to efficiently assess the primary and interaction effects of temperature and exposure length on the responses under investigation, while reducing the total number of experimental trials. Replication was mostly utilized to quantify pure experimental error and evaluate model adequacy, rather than to enhance the power of particular pairwise comparisons.

## 3. Results

### 3.1. Visual Assessment and Mass Variation

**Figure 1:**
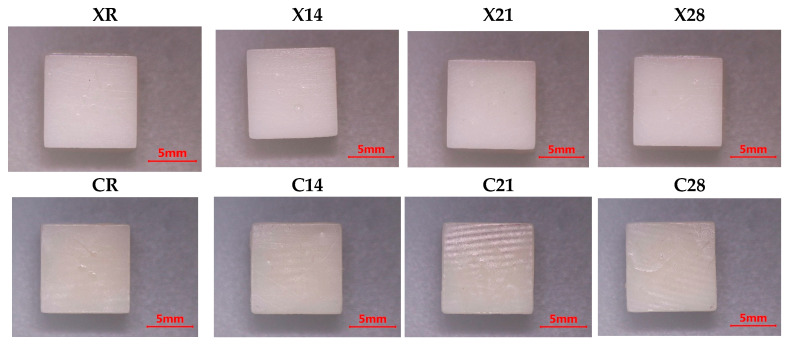
Visual aspect of Anycubic (X) and NextDent Creo (C) specimens after immersion in alcohol-based mouthwash. Group labels are defined according to the experimental design described in Section 2.2.

Following immersion in the alcohol-based mouthwash, neither material exhibited any noticeable color changes (Figure 1). The specimens maintained their original visual appearance during the 28-day duration.

**Figure 2:**
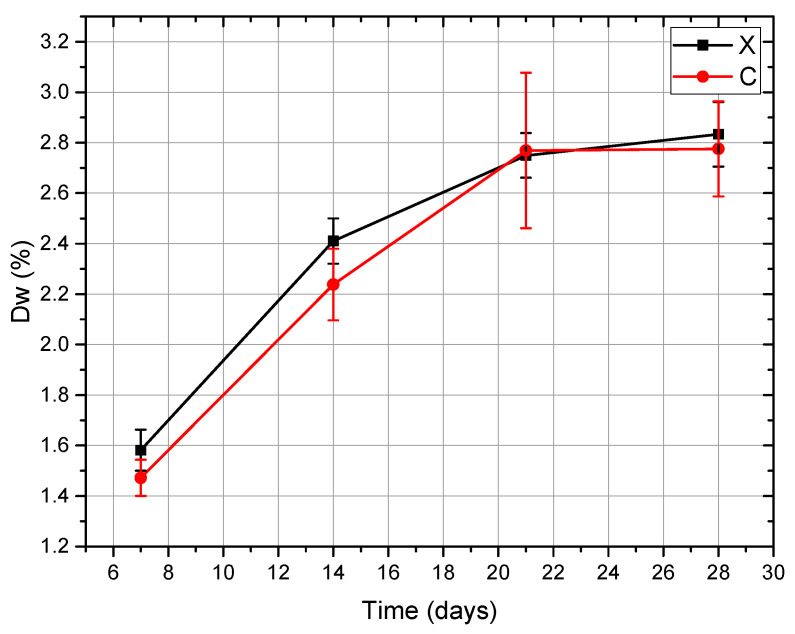
Mass variation (%) of Anycubic (X) and NextDent Creo (C) specimens during immersion in mouthwash, showing a progressive increase in mass with prolonged immersion time for both materials.

The mass variation study (Protocol A) indicated a progressive increase in mass across immersion time. For the Anycubic series, the variation followed the polynomial relationship (Figure 2), Equations (1) and (2), respectively:

(1) $$\Delta w=-0.0038,t^{\left\{2\right\}}+0.1916,t+0.4384$$

and for NextDent Creo:

(2) $$\Delta w\;=\;-0.0039,\;t^{\left\{2\right\}}+\;0.1992,\;t\;+\;0.2529$$

### 3.2. Contact Angle Analysis

#### 3.2.1. Water

The water contact angle of the Anycubic specimens in Protocol A substantially decreased after 21 days (*p* < 0.05), whereas the NextDent Creo samples exhibited a continuous decrease from the beginning, suggesting an increasingly hydrophilic surface.

**Figure 3:**
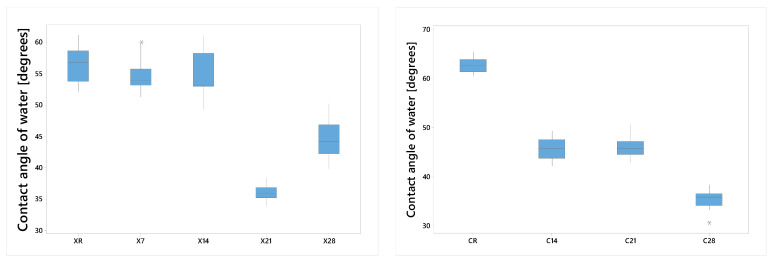
Water contact angle variation with immersion time for Anycubic (X) and NextDent Creo (C), indicating a time-dependent decrease in wettability during chemical aging. Group labels are defined according to the experimental design described in Section 2.2.

The mean contact angle of the reference specimens in Protocol B was 51.47 ± 1.69°. Following a 48-h aging period at 40 °C, the surface exhibited temporary hydrophobicity, characterized by an angle of 73.90 ± 1.83°, before progressively reverting to hydrophilic properties. Following 72 h at 60 °C (6072), the contact angle (52.26 ± 0.67°) was nearly identical to that of the reference. ANOVA revealed substantial differences across the groups, but Tukey/Fisher tests established a statistical equivalence between 0000 and 6072 (Figure 3).

The derived response surface model in coded units is θ_water = 61.81 − 4.576 T − 6.242 t + 1.271 Tt, exhibiting a maximum absolute inaccuracy of around 5%.

#### 3.2.2. Diiodomethane

**Figure 4:**
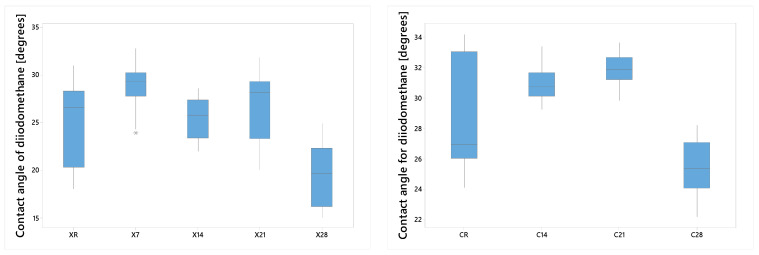
Diiodomethane contact angle as a function of immersion time (Protocol A), showing a non-linear response characterized by an initial increase followed by a decrease with prolonged immersion time, indicating changes in surface dispersive interactions. Group labels are defined according to the experimental design described in Section 2.2.

The diiodomethane contact angle exhibited a parabolic design in Protocol A, characterized by an initial increase followed by a decline. The ANOVA test demonstrated substantial treatment effects, although the Tukey and Fisher tests confirmed equivalence among XR, X14, X21, and, individually, X7 = X21 (Figure 4).

A significant reduction was observed in NextDent Creo after 28 days, with C14 and C21 demonstrating statistical equivalence.

In Protocol B, the reference value (25.19 ± 1.43°) rose to 27.63 ± 1.69° and 30.66 ± 1.80° for 4048 and 4072, respectively, but decreased to 18.80° for 6072. The RSM equation θ_DI = 26.23 − 2.914 T − 1.503 t − 3.017 Tt emphasizes temperature and the T × t interaction as predominant factors, where T represents the aging temperature (°C), and t denotes the exposure duration (h).; yet, the model error attained 18% at the center point, indicating a non-planar response surface.

#### 3.2.3. Ethylene Glycol

**Figure 5:**
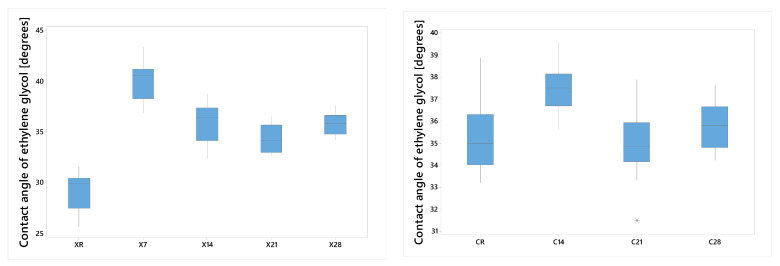
Ethylene glycol contact angle as a function of immersion time (Protocol A), initial increase followed by a gradual decrease or stabilization with immersion time, suggesting moderate changes in surface polar interactions. Group labels are defined according to the experimental design described in Section 2.2.

In Protocol A, Anycubic samples exhibited an initial rise in contact angle, followed by a constant reduction; ANOVA indicated significant effects (*p* < 0.05). For NextDent Creo, the results were comparatively constant, with CR, C21, and C28 exhibiting statistical equivalence (Figure 5).

Protocol B revealed a significant rise for 4048, presumably attributable to surface relaxation and alterations in micro-roughness. The RSM model θ_EG = 37.68 − 3.670 T − 1.196 t + 3.318 Tt accurately predicted experimental data within a 1% margin of error, validating a flat variation.

### 3.3. Surface Free Energy (SFE)

**Figure 6:**
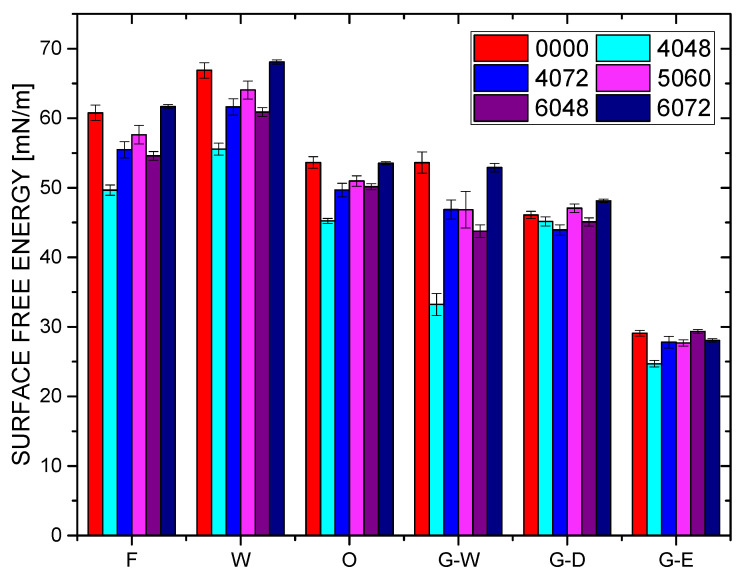
Surface free energy variation for PMMA samples under chemical (Protocol A) and thermal (Protocol B) aging, showing minimal variation during chemical aging and a gradual increase with immersion duration under thermal aging conditions.

The SFE values were obtained using the four conventional models. In Protocol A, the SFE remained almost constant during XR–X14 and thereafter rose, mirroring the pattern observed in the water contact angle. NextDent Creo samples demonstrated a gradual rise in surface free energy with prolonged immersion duration (Figure 6).

In comparison, Protocol B demonstrated a reduction in SFE with increasing temperature and exposure duration, with a minimum at 4048, thereafter recovering to near-reference or slightly elevated levels at 72 h and higher temperatures. The calibrated model, SFE = 49.65 + 2.200 T + 1.961 t − 0.2618 Tt, had a residual error of around 3%.

### 3.4. Shore D Hardness

In Protocol A, hardness diminished gradually with immersion duration. For the Anycubic set, the quadratic equation = 0.0053 t^2^ − 0.5243 t + 83.002 indicates the variation (82.89 ± 0.65 → 72.67 ± 2.08). For Next Dent Creo, the relationship ShD = 0.0106 t^2^ − 0.6446 t + 86.414 resulted in a progressive decrease from 86.56 ± 0.39 to 76.22 ± 2.25.

**Figure 7:**
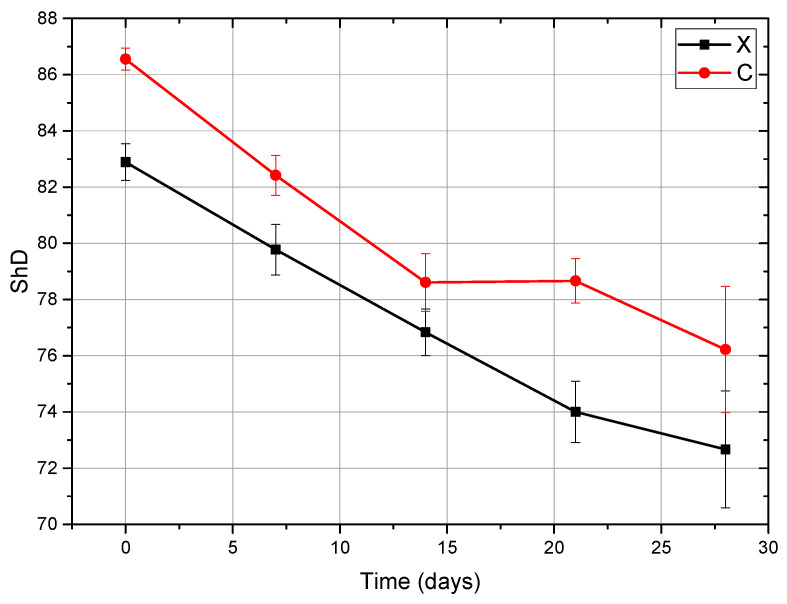
Shore D hardness vs. immersion time (Protocol A, Anycubic (X) and Next Dent (C)), demonstrating a gradual decrease in hardness with increasing immersion time for both PMMA materials.

ANOVA demonstrated significant effects, indicating that X21 and X28, as well as C14 and C21, are statistically equal (Figure 7).

**Figure 8:**
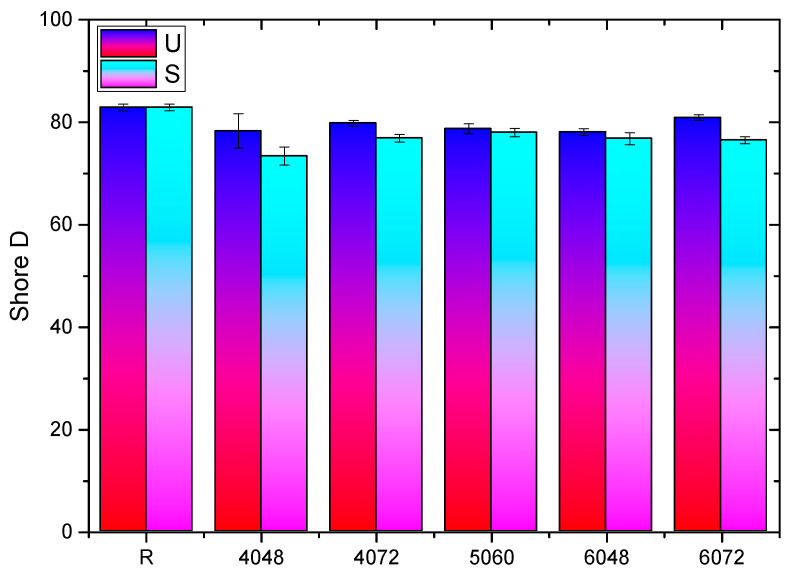
Shore D hardness (upper and lateral surfaces) vs. T–t (Protocol B).

In Protocol B, hardness fluctuated based on the testing surface and the heat conditions. Tukey’s test revealed equivalence between the reference and 6072 for the top surface (U), as well as among the sets 4072, 5060, 4048, and 6048. The lateral surface (S) exhibited equivalence among the sets 5060, 4072, 6072, and 6048, although the reference and 4048 displayed considerable differences (Figure 8).

Temperature had a negative correlation with hardness (r ≈ −0.71 for U and S), as did time (r = −0.55 for U; −0.70 for S). The response-surface equations were as follows: U: ShD = 79.27 + 0.225 T + 1.075 t + 0.325 Tt; S: ShD = 75.90 + 0.75 T + 0.80 t − 0.95 Tt.

The data indicate that time and the T × t interaction have the most significant impact.

### 3.5. Mechanical Properties Under Compression

**Figure 9:**
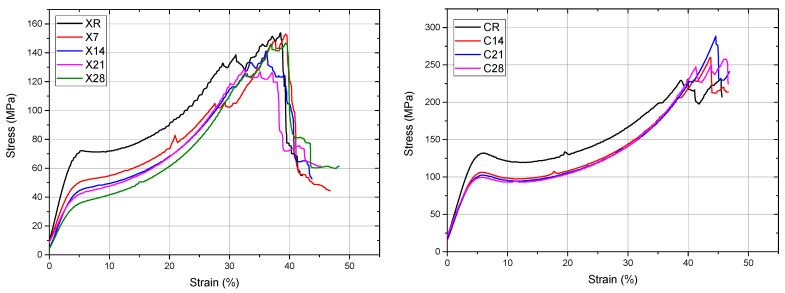
Stress–strain curves (as-printed vs. aged samples), illustrating reduced stiffness and the disappearance of a distinct yield point following aging. Group labels are defined according to the experimental design described in Section 2.2.

The stress–strain curves demonstrated flexible behavior for all materials, with a distinct peak in the as-printed condition that vanished post-aging (Figure 9). Both treatments resulted in a decrease in the elastic modulus and yield strength.

In Protocol A, the modulus decreased exponentially with time. For Anycubic, ANOVA (*p* < 0.05) and Tukey/Fisher tests demonstrated statistical equivalence between X14 = X21 and X21 = X28. In the NextDent Creo series, the reference constituted a separate category, although C14, C21, and C28 exhibited statistically equivalent means.

In Protocol B, the RSM model E = 1016 − 90.54 T − 4.767 t + 41.23 Tt accurately fitted the data with an approximate error of 5% and demonstrated modulus reductions ranging from 35% to 46% relative to the reference.

The yield strength exhibited similar tendencies. In Protocol A, X samples exhibited a consistent decline, while C samples stabilized after 14 days. For Protocol B, the fitted model YS = 37.74 − 1.209 T + 0.763 t + 0.572 Tt produced residuals under 2%, confirming temperature as the primary variable.

The stress at first failure and compressive strength exhibited significant standard deviations, undermining their statistical reliability; yet, both metrics typically diminished during aging.

### 3.6. Fractographic Observations

The fractographic analysis offers supplementary insights that corroborate the mechanical results of this investigation. The resemblance of fracture surface characteristics noted across the examined specimens suggests a uniform mechanical response to compressive pressure, mostly defined by brittle fracture behavior. This discovery indicates that the aging treatments did not cause significant surface plasticization that might modify the failure mechanism.

Furthermore, the lack of localized deformation zones and the existence of generalized fracture patterns suggest a reasonably equal stress distribution throughout the specimen’s cross-section. The findings validate the mechanical test results, affirming that the noted decreases in elastic modulus and yield strength indicate bulk material deterioration rather than surface-limited phenomena.

**Figure 10:**
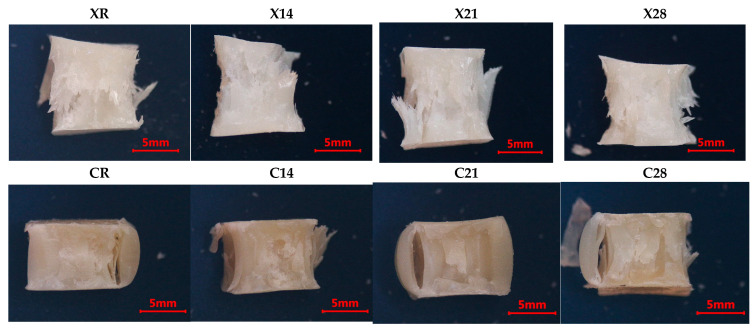
Fractographic analysis for protocol A. Group labels are defined according to the experimental design described in Section 2.2.

**Figure 11:**
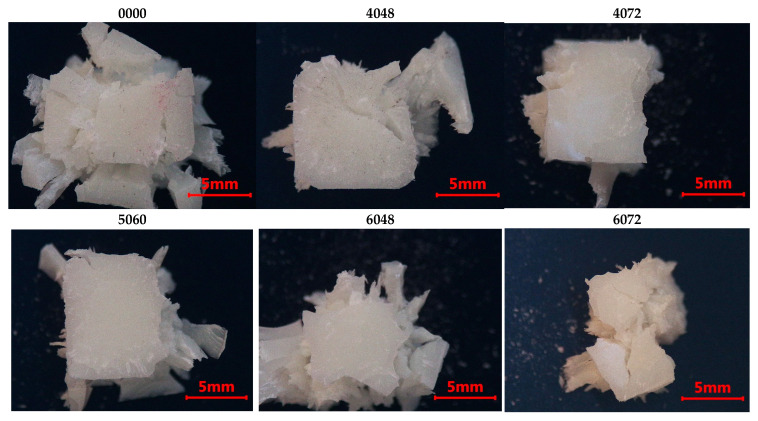
Fractographic analysis for protocol B. Group labels are defined according to the experimental design described in Section 2.2.

Microscopic examination showed radial fracture patterns with watercourse markings and localized fractures in all samples. The fracture mode shifted from pure compression to shear at roughly 45°, with no significant changes noted across the aging procedures. Specimens subjected to high temperatures in artificial saliva demonstrated reduced elasticity, resulting in more damaged fracture surfaces (Figure 10 and Figure 11).

### 3.7. Literature-Based Analysis of Mechanical Degradation of PMMA Under Aging Conditions

#### 3.7.1. Classification of PMMA Materials

Based on the data extracted from multiple publications, the studies were grouped according to the manufacturing process of the specimens used for testing (Table 2) [37,38,39,40]:

- Class 1: Heat-polymerized PMMA;
- Class 2: Cold-polymerized PMMA;
- Class 3: CAD/CAM-fabricated PMMA.

Polynomial regression equations (second-order) were derived from literature data for each class, providing the best correlation between immersion duration and compressive strength decrease [41,42].

The current research characterized accelerated aging behavior by unifactorial second-order polynomial regressions and two-factor response surface models. Table 3 illustrates and compares these polynomial models with regression-based deterioration models documented in the literature for PMMA materials.

#### 3.7.2. Regression Analysis and Comparative Findings

A graphical representation of the fitted curves demonstrates the temporal decrease in compressive strength across the three material categories.

**Figure 12:**
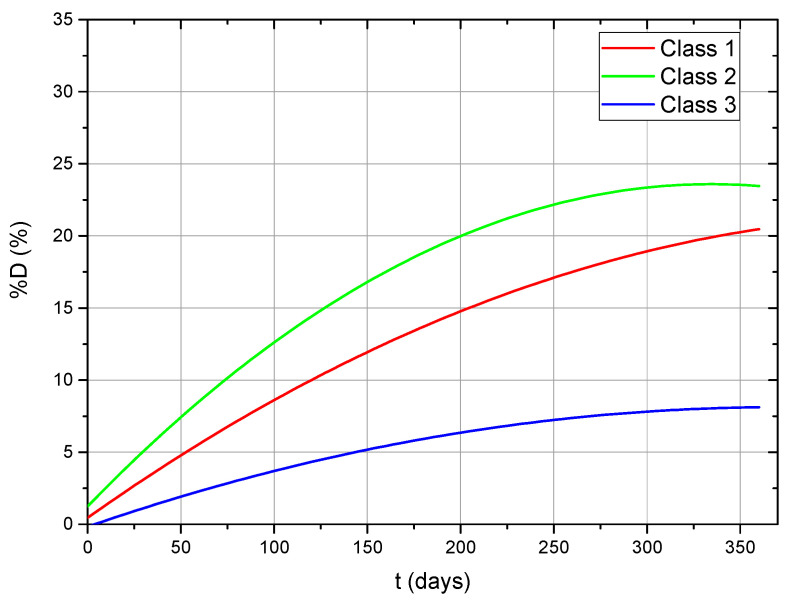
Percentage decrease in compressive strength with increasing immersion time for PMMA materials of different fabrication types.

The statistics suggest that cold-polymerized PMMA displayed the highest degradation rate, whereas CAD/CAM-produced PMMA showed a minimal reduction in compressive strength over time (Figure 12). The findings indicate that the polymerization method and conversion rate substantially affect long-term structural integrity [43,44].

Accelerated aging experiments, correlating temperature and time, indicate that a compressive strength reduction of approximately 15% after 72 h of immersion at elevated temperatures corresponds to roughly 205 days of natural immersion for heat-polymerized PMMA (Class 1) and 127 days for cold-polymerized PMMA (Class 2). For CAD/CAM PMMA (Class 3), deterioration maintained under 10%, preventing direct comparison with natural circumstances [45,46,47,48].

Samples immersed in mouthwash solutions demonstrated a non-linear deterioration trend, with turning points dependent on immersion duration. The phenomenon indicates the existence of unique degradation pathways in contrast to artificial saliva, perhaps resulting from solvent infiltration and polymer chain relaxation triggered by alcohol-based constituents. The maximum recorded deterioration (≈12%) occurs after roughly 151 days for Class 1 materials and 94 days for Class 2 materials.

#### 3.7.3. Mechanical and Clinical Analyses

While compressive strength is an important metric for assessing mechanical performance, it serves as a late-stage indicator of material deterioration, as fracture transpires only after significant structural weakening. In contrast, the elastic modulus serves as a more sensitive indicator of initial degradation. Longer immersion results in a statistically significant reduction in elastic modulus, which is directly associated with raised deformation and diminished stiffness [49,50,51,52,53,54].

Clinically, this condition may present as the patient’s sense that the temporary restoration “softens” or “deteriorates” over time. Furthermore, the corresponding reduction in yield strength indicates that plastic deformation may transpire under unintentional loading, thus jeopardizing the integrity or efficacy of long-term interim restorations.

## 4. Discussion

The purpose of the present study was to examine how the surface and mechanical performance of 3D-printed PMMA provisional materials were affected by simulated oral environments, particularly thermal and chemical aging. The results indicated that both temperature and immersion in alcohol-based solutions substantially influenced the surface free energy, hardness, and compressive strength of printed PMMA, while the extent and nature of these effects ranged among materials and situations.

In artificial saliva, thermal exposure resulted in a moderate, time-dependent change in wettability [55]. The initial rise in contact angle at lower temperatures may indicate a temporary reorganization of polymer chains, resulting in less polarity at the surface layer [56]. Prolonged exposure at 60 °C or longer immersion periods favored the development of a more hydrophilic surface, aligning with the migration of polar groups and potential hydrolytic rupture of side chains [57].

These findings align with the data of Gad et al. [58], who indicated that changes in salivary pH and temperature stimulate hydrolytic degradation and surface softening in PMMA materials used for removable prostheses. The current investigation concentrated on provisional restoration; however, similar degradation mechanisms might occur through water absorption and chain softening.

Chemical aging in alcohol-containing mouthwash intensified the surface effects. Both resins exhibited reduced contact angles and higher total surface free energy, signifying improved surface polarity and a shift towards hydrophilicity. Ethanol likely promoted the diffusion of solvent molecules into the polymer matrix, leading to micro-swelling and partial extraction of low-molecular-weight constituents. This process is characterized as a mixed plasticization–oxidation mechanism, often linked to diminished gloss and heightened roughness during prolonged exposure [59,60,61]. Among the evaluated materials, NextDent Creo demonstrated superior surface stability, likely because of its higher cross-linking density and precise curing parameters characteristic of medical-grade formulations.

When immersed in mouthwash, the Shore D hardness gradually decreased, which is consistent with the plasticization behavior of methacrylate polymers in water–alcoholic conditions. Lahoti et al. [6] published the same findings, demonstrating that exposure to acidified saliva substantially decreased both the flexural and impact strength of heat polymerized PMMA, with structural degradation happening even after short immersion durations. The present findings indicate that 3D-printed PMMA materials are susceptible to this degradation process, yet the rate of softening and modulus drop varies according to resin type. The Anycubic exhibited a more pronounced drop, presumably attributable to its reduced polymerization temperature and higher residual monomer content, which facilitates solvent absorption and chain mobility.

This interpretation was corroborated by the compressive stress–strain curves, which demonstrated that the aged samples evidenced reduced rigidity and softer yielding, which indicates a decrease in intermolecular cohesion [1]. These behaviors align with the hydrolytic and solvent-induced relaxation processes outlined in other research on PMMA and CAD/CAM polymers [62]. Researchers observed that water and ethanol might function as mild solvents that infiltrate polymer chains, resulting in volumetric expansion and a noticeable reduction in mechanical stiffness [63,64].

Despite the effects above, both evaluated materials maintained sufficient structural integrity for short-term temporary uses. This indicates that, while chemical aging may modify the surface and mechanical characteristics of printed PMMA, the extent of deterioration throughout standard clinical service durations (days to weeks) remains clinically acceptable. In a comparative analysis of the two materials and the two exposure protocols, it is obvious that thermal aging primarily affects surface polarity and energy, while chemical aging affects both surfaces and bulk mechanical properties [65,66]. NextDent Creo consistently showed superior resistance to both forms of degradation, likely due to its increased filler content and polymerization conversion [67]. In contrast, Anycubic Basic White, designed for ordinary 3D printing, had increased susceptibility to temperature and alcohol, suggesting that non-medical photopolymers may be less appropriate for prolonged intraoral application [68].

Provisional restorations may be more susceptible to staining, plaque accumulation, and faster wear in patients who routinely use alcohol-based mouthrinses or have acidic salivary pH, because of increased surface free energy and reduced hardness during clinical examinations [69]. Consequently, the choice of a high-density, cross-linked PMMA resin is essential when prolonged use of temporary restorations is expected [70,71,72].

The noted distinctions between chemical and thermal aging procedures may offer clinical advice for the selection of temporary materials in particular patient situations. The current study, although performed in vitro, indicates that extended contact with alcohol-based oral hygiene products may lead to progressive softening and mechanical deterioration of specific 3D-printed PMMA materials. Consequently, for patients who often utilize alcohol-based mouthwashes, provisional materials exhibiting increased resistance to solvent-induced plasticization and superior polymerization conversion may be useful, especially when prolonged temporary usage is expected [56].

In contrast, the significant impact of thermal aging on elastic modulus and yield strength suggests that temperature-induced stress may be crucial for long-term mechanical stability. Patients with acidic salivary conditions, frequent use of hot drinks, or parafunctional behaviors may benefit from provisional materials that demonstrate improved thermal stability and retention of stiffness [73]. Given these limitations, the current findings suggest a more personalized strategy in preliminary material selection, including patient-specific risk factors and the expected duration of intraoral use.

The results highlight the need for storage and cleaning guidelines for temporary crowns made from 3D-printed PMMA. Minimizing extended contact with alcohol-based solutions and preserving neutral salivary environments may substantially improve their dimensional and aesthetic stability [74,75].

## 5. Conclusions

Chemical and thermal aging influence the physical and mechanical properties of both 3D-printed PMMA materials taken into the study. Therefore, printed PMMA resins exhibit dimensional and mechanical stability during short-term exposure; however, extended contact with alcohol-based substances or high temperatures may compromise their surface characteristics and mechanical strength.

Exposure to alcohol-based mouthwash resulted in a considerable mass increase, decreased hardness, and increased surface free energy, indicating higher surface hydrophilicity. Thermal aging in artificial saliva caused a brief hydrophobic change, subsequently followed by recovery, mostly influenced by exposure duration.

Of the two protocols used (maintenance in alcohol-based mouthwash and thermal aging in artificial saliva), the second one was more effective in accelerating the degradation of the investigated 3D-printed PMMA materials. After maintenance in alcohol-based mouthwash, the SFE remained almost constant for all samples, mirroring the pattern observed in the water contact angle. After thermal aging in artificial saliva, all samples demonstrated a gradual rise in surface free energy with prolonged immersion duration. In this case, a reduction in SFE was observed with increasing temperature and exposure duration, with a minimum at 48 h maintenance at 40 °C, thereafter recovering to near-reference or slightly elevated levels at 72 h and higher temperatures.

The elastic modulus and yield strength were identified as the most dependable mechanical parameters, exhibiting reductions of up to about 46% compared to as-printed standards. Fracture morphology remained uniform; however, elevated temperatures facilitated more fragmented failure surfaces.

The chemical aging protocol employed a single alcohol-based mouthwash and therefore represents a simplified model of oral chemical exposure.

The current study offers valuable insights into the environmental stability of printed PMMA; however, it is an in vitro evaluation. The intricate synergistic interactions of salivary enzymes, temperature variations, and masticatory stress may expedite breakdown in vivo.

It should be noted that this study has several limitations in addition to its in vitro character. The aging procedures were implemented separately, so the possible synergistic effects of concurrent chemical and thermal stresses, which may arise in clinical settings, were not assessed. The accelerated aging periods were intentionally brief and aimed at producing quantifiable alterations within a regulated experimental timescale, rather than accurately simulating prolonged intraoral service conditions. These limitations must be acknowledged when interpreting the data, and future research should try to integrate combined aging procedures and prolonged exposure durations to replicate complex oral environments more accurately.

The cubic geometry of the examined specimens contrasts with the complex form and stress distribution of clinical temporary crowns. Standardized specimen geometries are essential for the reliable characterization of intrinsic material characteristics. Both cylindrical and prismatic specimens can be used for compressive testing of polymeric materials in accordance with ASTM D695 [76] and ISO 604 standards [77].

This study deliberately chose a standardized shape to guarantee repeatability and for relevant comparisons of material behavior under controlled settings. The aim was to assess alterations in the inherent mechanical characteristics of the 3D-printed PMMA materials, rather than to duplicate the stress distribution of clinical restorations. Subsequent research will broaden this inquiry to specimen shapes that more accurately reflect possible crown designs to evaluate structure-dependent mechanical performance.

Future research should explore combined thermo-mechanical aging protocols and investigate potential reinforcement strategies—such as nanoparticle incorporation or surface coatings—to improve the durability of 3D-printed provisional materials. Additionally, spectroscopic and microscopic analyses would be beneficial for elucidating chemical changes at the molecular level.

## Figures and Tables

**Table 1:** Chemical composition of Fusayama-type artificial saliva.

Component	Chemical Formula	Concentration (g·L^−1^)
Sodium chloride	NaCl	0.40
Potassium chloride	KCl	0.90
Urea	CO(NH_2_)_2_	1.00
Sodium dihydrogen phosphate	NaH_2_PO_4_	0.69
Calcium chloride dihydrate	CaCl_2_·2H_2_O	0.795
Sodium sulfide nonahydrate	Na_2_S·9H_2_O	0.005

**Table 2:** Polynomial regression equations and R^2^ values for each PMMA group.

Group	Equation	R^2^
Class 1	$\%D=-0.0001t^{2}+0.0916t+0.452$	0.9911
Class 2	$\%D=-0.0002t^{2}+0.1337t+1.2447$	0.9845
Class 3	$\%D=-(6\times 10^{-5})t^{2}+0.0446t-0.1649$	0.9981

**Table 3:** Comparison of polynomial degradation models used in the present study and reported in the literature for PMMA materials.

PMMA Type/Class	Aging Protocol	Independent Variables	Property Modeled	Polynomial Model Type	Key Observation
3D-printed PMMA (Anycubic, NextDent)	Chemical aging (mouthwash)	Time (t)	Mass variation (Δw)	2nd-order polynomial (t^2^)	Non-linear solvent uptake with time
3D-printed PMMA	Chemical aging (mouthwash)	Time (t)	Shore D hardness	2nd-order polynomial (t^2^)	Progressive softening due to plasticization
3D-printed PMMA	Thermal aging (artificial saliva)	Temperature (T), time (t)	Contact angle (θ_water, θ_DI, θ_EG)	RSM (2nd-order polynomial with T·t)	Wettability primarily governed by temperature
3D-printed PMMA	Thermal aging (artificial saliva)	Temperature (T), time (t)	Surface free energy	RSM (2nd-order polynomial with T·t)	Combined thermal–time interaction
3D-printed PMMA	Thermal aging (artificial saliva)	Temperature (T), time (t)	Elastic modulus, yield strength	RSM (2nd-order polynomial with T·t)	Accelerated stiffness and strength degradation
Class 1 PMMA (heat-polymerized)	Saliva immersion	Time	Compressive strength	2nd-order polynomial	Moderate long-term degradation
Class 2 PMMA (cold-polymerized)	Saliva immersion	Time	Compressive strength	2nd-order polynomial	Highest degradation rate
Class 3 PMMA (CAD/CAM)	Saliva immersion	Time	Compressive strength	2nd-order polynomial	Minimal degradation over time

## Data Availability

The original contributions presented in the study are included in the article. Further inquiries can be directed to the corresponding authors.

## Abbreviations

The following abbreviations are used in this manuscript:

SFE	Surface free energy
PMMA	Polymethyl methacrylate
CAD/CAM	Computer-aided design/computer-aided manufacturing
ANOVA	Analysis of variance
RSM	Response surface methodology
ShD/Shore D	Shore D hardness
YS	Yield strength
SPSS	Statistical Package for the Social Sciences
OWKR	Owens–Wendt–Rabel–Kaelble
DI	Diiodomethane
EG	Ethylene glycol
